# Sheng Mai San Modulates the Heart-Gut-Microbiota Axis to Mitigate Heat Stress-Induced Damage in Rats

**DOI:** 10.3390/life15060841

**Published:** 2025-05-23

**Authors:** Jiaqi Dong, Xiaoli Li, Wei Xiao, Xiaosong Zhang, Peng Ji, Yanming Wei

**Affiliations:** 1 College of Veterinary Medicine, Gansu Agricultural University, Lanzhou 730070, China; 18893811442@163.com (J.D.); lxl15006995983@163.com (X.L.); 13340923219@163.com (W.X.); jip@gsau.edu.cn (P.J.); 2 College of Veterinary Medicine, China Agricultural University, Beijing 100091, China; zhangxsgs@163.com

**Keywords:** Sheng Mai San, heat stress, cardiac injury, small intestine injury, gut microbiota

## Abstract

Heat stress has become a significant challenge in animal husbandry and human health, posing significant threats to both livestock and human health and profoundly impacting agricultural productivity. Sheng Mai San has been shown to effectively alleviate heat stress, yet the underlying mechanisms remain unclear. Therefore, this study established a heat stress model and employed Sheng Mai San as an intervention, with NAC as the positive control. Using histopathological analysis, Western blotting, ELISA, and 16S rDNA sequencing, we investigated the protective effects of Sheng Mai San against heat-stress-induced cardiac and intestinal injuries, as well as gut microbiota dysbiosis. The results demonstrated that heat stress-induced cardiac injury primarily occurred within 6–12 h of the cessation of heat stress. This injury was manifested by a significant elevation in the cardiac index, accompanied by attenuated expression of cardiac antioxidants (GSH, SOD, CAT, and T-AOC) and increased MDA content. Following Sheng Mai San intervention, the cardiac index was reduced, antioxidant indices (GSH, SOD, and CAT) were significantly elevated, and MDA and inflammatory markers (IL-1β, IL-6, and TNF-α) were markedly decreased. Additionally, Sheng Mai San was found to activate the Keap1-Nrf2 signaling pathway in the heart. Sheng Mai San demonstrated significant protective effects on small intestinal morphology, attenuating pathological alterations while promoting goblet cell proliferation. Analysis of the gut microbiota revealed that Sheng Mai San increased the Chao1, ACE, Shannon, and Simpson indices while reducing the abundance of harmful bacteria, such as *g_Globicatella*, *g_Thermoactinomyces*, *g_Staphylococcus*, *g_Gemella*, and *g_Veillonella*. Additionally, it promoted the expression of beneficial bacteria, including *g_Lactobacillus* and *g_Ruminococcaceae*. In summary, Sheng Mai San alleviates heat stress-induced cardiac hypertrophy and restores the oxidative stress balance in the heart. It also mitigates pathological damage in the small intestine, enhances the diversity and richness of the gut microbiota, and ameliorates gut microbiota dysbiosis. These findings highlight the significance of the heart-small intestine-gut microbiota axis in the protective effects of Sheng Mai San against heat stress injury. This study provides a potential therapeutic approach for heat-stress-related diseases and offers insights into the development of anti-heat-stress drugs.

## 1. Introduction

Heat stress (HS) is a critical environmental stressor that impacts human and animal well-being globally [1], with particularly pronounced effects in tropical and subtropical climatic zones [2]. This complex physiological challenge arises from the synergistic interaction of multiple environmental parameters, predominantly encompassing elevated ambient temperature, increased relative humidity, solar radiation intensity, and airflow velocity. Among these, high temperature and high humidity are the main factors influencing the heat stress response. Environmental conditions characterized by high temperatures and humidity can lead to reduced reproductive performance, diminished animal welfare, decreased fertility, increased susceptibility to diseases, and, in extreme cases, elevated mortality rates [3]. Heat stress can also trigger physiological responses in animals, such as reduced feed intake, water loss, body weight reduction, elevated rectal temperature, and increased respiratory and heart rates [4]. However, the molecular mechanisms underlying the perception and regulation of temperature fluctuations are still unclear.

The heart is a highly heat-stress-sensitive organ. Under heat stress, excessive reactive oxygen species (ROS) are generated in the hearts of animals. The overproduction of ROS continuously attacks macromolecules, such as nucleic acids, proteins, and lipids, within cardiomyocytes, disrupting their structure and function while also inhibiting the biological activity of antioxidant enzymes. This leads to oxidative stress damage in cardiomyocytes and, in severe cases, may even result in cardiomyocyte apoptosis and necrosis [5]. Nuclear factor erythroid 2-related factor 2 (Nrf2) is a pivotal transcriptional regulator that governs cellular redox balance and constitutes a fundamental defense mechanism against oxidative damage. Under normal physiological conditions, Nrf2 forms a complex with Kelch-like ECH-associated protein 1 (Keap1) in the cytosol, undergoing subsequent degradation through the ubiquitin-proteasome system mediated by Keap1, thereby maintaining low levels of expression. However, under oxidative stress conditions, elevated levels of ROS oxidize key cysteine residues in Keap1, leading to the inactivation of the Keap1-Cul3 complex and thereby inhibiting the ubiquitination and degradation of Nrf2 [6]. Subsequently, Nrf2 undergoes nuclear translocation and associates with antioxidant response elements (AREs), thereby activating the expression of downstream genes encoding crucial antioxidant enzymes, such as glutathione peroxidase (GPx), superoxide dismutase (SOD), and catalase (CAT) [7]. Hayes et al. demonstrated that activation of the Keap1-Nrf2 signaling pathway upregulates the expression of GSH (Glutathione), SOD, and CAT [8].

Heat stress significantly affects the small intestine (duodenum, jejunum, and ileum) and gut microbiota in rats. Studies have shown that heat stress triggers significant architectural remodeling of the intestinal morphology, manifesting as marked villus atrophy and concurrent crypt hyperplasia in the duodenal mucosa. These structural alterations are accompanied by the induction of oxidative stress and inflammatory responses, ultimately compromising the intestinal digestive capacity and nutrient absorption efficiency [9]. In the jejunum, heat stress suppresses the activity of digestive enzymes (such as lactase and sucrase), reduces the efficiency of nutrient digestion, and induces epithelial cell apoptosis, further compromising its absorptive capacity. Furthermore, heat stress significantly inhibits the secretory function of goblet cells, leading to thinning of the intestinal mucus layer and weakening of its physical barrier against pathogenic bacteria [10]. Goblet cells are a critical component of the intestinal epithelium and are responsible for secreting mucus to form the mucus layer, which protects the intestinal epithelium from pathogens and toxins [11]. Heat stress reduces both the number and function of goblet cells, resulting in decreased mucus layer thickness. This increases the likelihood of direct contact between pathogens and epithelial cells, further exacerbating intestinal inflammation and damage [10]. In the ileum, heat stress manifests as a reduction in beneficial bacteria (such as *Lactobacillus* and *Bifidobacterium*) and an increase in opportunistic pathogens (such as *Salmonella*), leading to microbial dysbiosis and localized inflammatory responses [12]. Additionally, heat stress significantly reduces gut microbiota diversity and inhibits the production of short-chain fatty acids (SCFAs), thereby weakening the protective effects of these bacteria on intestinal epithelial cells [13,14,15]. Therefore, mitigating the negative impacts of heat stress on the small intestine and gut microbiota may be a crucial strategy for improving overall health.

Modern medical research has shown that the heart supplies oxygen and nutrients to the small intestine through blood circulation, while the nutrients absorbed by the small intestine enter the liver via the portal vein system and are subsequently distributed throughout the body. Cardiac dysfunction may reduce intestinal blood flow, leading to intestinal ischemia and impaired barrier function, which can result in bacterial and endotoxin translocation, exacerbating systemic inflammation and increasing the cardiac burden [16]. Tang et al. found that myocardial infarction may trigger a systemic inflammatory response, impair intestinal barrier function, and increase intestinal permeability. The entry of intestinal endotoxins into the bloodstream may further aggravate cardiac inflammation and injury [17]. The gut microbiota, as endogenous microorganisms of the small intestine, is closely associated with small intestinal dysfunction. Gut microbiota dysbiosis can impair the small intestine’s ability to properly “transform and transport” nutrients, thereby affecting blood circulation and contributing to the development of cardiovascular diseases [18].

Currently, the common approaches to managing heat stress include rapid cooling, fluid replacement, and pharmacological interventions [19]. Studies have shown that effective antioxidants can mitigate heat-stress-induced damage. However, current strategies for mitigating heat stress are constrained, and effective therapeutic interventions are notably deficient. Therefore, identifying effective natural remedies to prevent and alleviate heat stress is crucial. Sheng Mai San (SMS), a classic formulation for replenishing qi and nourishing yin, is clinically used to treat critical conditions such as heatstroke, heart failure, liver failure, and shock, as well as chronic diseases like chronic bronchitis, tuberculosis, and diabetes, demonstrating favorable therapeutic outcomes. However, the mechanisms underlying the effects of Sheng Mai San on heat stress-induced cardiac and intestinal injuries and gut microbiota dysbiosis remain unclear. Therefore, this study established a heat stress model to investigate the regulatory mechanisms of Sheng Mai San on the heart-small intestine-gut microbiota axis under heat stress.

## 2. Materials and Methods

### 2.1. Reagents

The BCA protein concentration assay kit (Catalog No.: PC0020) and tissue cytoplasm/nucleus separation kit (Catalog No.: EX2660) were purchased from Solarbio (Beijing, China). CAT (Catalog No.: A007-2-1), SOD (Catalog No.: A001-3-2), GSH (Catalog No.: A006-2-1), T-AOC (Total Antioxidant Capacity) (Catalog No.: A015-2-1), and MDA (Malondialdehyde) (Catalog No.: A003-1-2) assay kits were obtained from Nanjing Jiancheng Bioengineering Institute (Nanjing, China). Antibodies against Nrf2 (Catalog No.: 16396-1-AP) and Keap1 (Catalog No.: 10503-2-AP) were acquired from Proteintech (Wuhan, China). Enzyme-linked immunosorbent assay (ELISA) kits for inter-leukin-1β (IL-1β), interleukin-6 (IL-6), and tumor necrosis factor-α (TNF-α) were purchased from Shanghai Enzyme-linked Biotechnology Co., Ltd. (Shanghai, China).

### 2.2. Preparation of Sheng Mai San

Sheng Mai San was prepared following standardized traditional Chinese medicine decoction protocols as previously described [20,21]. The herbal components of Sheng Mai San (*Panax ginseng C.A. Mey*., *Ophiopogon japonicus* (*L.f.*) *Ker-Gawl*., and *Schisandra chinensis* (*Turcz.*) *Baill*.) in a 3:3:2 ratio were pulverized and sieved through a 20-mesh screen, followed by soaking in 10 volumes of distilled water for 30 min. The mixture was decocted by rapid boiling and subsequent simmering for 60 min, filtered, and then refluxed with 8 volumes of distilled water for 40 min. After combining the filtrates, the solution was centrifuged at 3000 rpm for 15 min, concentrated under reduced pressure at 60 °C to 1.0 g crude herb/mL, and lyophilized to obtain the final freeze-dried powder.

### 2.3. Establishment of a Rat Model of Heat Stress

Forty-eight male SPF-grade healthy Sprague-Dawley (SD) rats (body weight 200 ± 20 g) were obtained from the Lanzhou Veterinary Research Institute of the Chinese Academy of Agricultural Sciences [Laboratory Animal License No.: SCXK (Gan) 2020-0002] and acclimatized for 7 days in a standard facility (temperature 23 ± 1 °C, relative humidity 45 ± 5%, 12 h/12 h light/dark cycle) with free access to food and water. Prior to the experiments, baseline body weight was recorded, and core temperature was measured using a rectal thermometer (inserted 2 cm deep). The heat stress (HS) model was established according to a previously published protocol [22]. Rats were exposed to 38 ± 1 °C and 75 ± 5% relative humidity in an artificial climate chamber for 2 h daily (10:00–12:00) for 7 consecutive days, with food and water deprivation during HS sessions. Body weight and temperature were measured immediately after each HS session before returning the animals to the standard conditions. Following the final HS exposure, samples were collected at 0, 3, 6, 9, and 12 h time points under anesthesia induced by intraperitoneal injection of 2% sodium pentobarbital.

### 2.4. Drug Intervention Treatment with Sheng Mai San

Sixty SD rats were divided into six groups and administered treatments by gavage 2 h before exposure to daily heat stress. The control and HS groups were administered a normal saline solution daily. The dosage of Sheng Mai San for rats was calculated based on human clinical equivalent dose conversion using the formula Dr = (Dh/W) × F, where Dr represents the rat dose (g/kg), Dh is the human clinical dose (g), W is the standard human body weight (60 kg), and F is the species conversion factor (6.3) for rat-to-human dose translation according to body surface area normalization principles. The Sheng Mai San intervention groups were administered SMS-H (5.04 g/kg), SMS-M (2.52 g/kg), and SMS-L (1.26 g/kg). Additionally, a positive control group received N-acetylcysteine (NAC) (150 mg/kg) [23]. The experimental rats were administered the treatment once a day for 7 days. The control group was maintained at an ambient temperature of 23 ± 1 °C and relative humidity of 45 ± 5%. The heat stress model and treatment groups were subjected to heat stress conditions for 7 consecutive days, as per the established model, with sampling conducted 6 h after the final heat stress session.

### 2.5. Behavioral and Physiological Observations in Rats

Behavioral changes in rats were observed before and after exposure to heat stress. Body weight and body temperature were recorded for each group before and after heat stress, along with daily food intake and the amount of water consumed.

### 2.6. Measurement of Heart Index

After collecting blood from the abdominal aorta, the heart was quickly excised, the surface moisture was absorbed, and the heart weight was recorded. The cardiac index was calculated as the ratio of the whole heart weight (HW) to the body weight (BW).

### 2.7. Determination of CAT, SOD, GSH, T-AOC, and MDA Levels in the Heart

Heart tissue was collected, rinsed with PBS, immediately snap-frozen in liquid nitrogen, and preserved at −80 °C for further biochemical analysis. A 10% heart tissue homogenate was prepared and centrifuged at 3000× *g* for 15 min at 4 °C. The protein concentration of the samples was determined using a BCA assay kit. The cardiac tissue concentrations of CAT, SOD, GSH, T-AOC, and MDA were quantified according to the manufacturer’s protocols for the respective commercial assay kits.

### 2.8. Determination of IL-1β, IL-6, and TNF-α Levels in the Heart

The concentrations of IL-1β, IL-6, and TNF-α in the cardiac tissue were determined in strict accordance with the manufacturer’s protocols provided with the respective assay kits.

### 2.9. Western Blotting Analysis

#### 2.9.1. Determination of Total Protein Content in the Heart

Total protein was extracted from rat heart tissue using RIPA lysis buffer, and the protein concentrations were determined using a BCA assay kit. The loading amounts for each sample were subsequently calculated based on the quantified protein concentrations. SDS-PAGE gels were prepared, and electrophoresis was performed at 80 V for 40 min, followed by 100 V for 80 min. Proteins were transferred to PVDF membranes at 200 mA for 90 min. The membranes were subjected to a 2 h blocking procedure at ambient temperature, subsequently incubated with specific primary antibodies targeting Keap1 (1:3000 dilution), Nrf2 (1:3000 dilution), and GAPDH (1:2000 dilution) at 4 °C for overnight incubation. The membranes were subjected to extensive washing with TBST for ten cycles, followed by incubation with horseradish peroxidase (HRP)-conjugated secondary antibodies at ambient temperature for 1 h, and subsequently washed thoroughly with TBST for an additional ten cycles. Protein bands were visualized using an ECL detection reagent, and images were captured. The relative expression levels of the target proteins were quantified by densitometric analysis using ImageJ software (v1.8.0; National Institutes of Health, Bethesda, MD, USA).

#### 2.9.2. Determination of Nuclear Protein Content in the Heart

Approximately 50 mg of heart tissue was weighed and homogenized in 500 μL of reagent A containing protease inhibitors at −10 °C for 20 min. The homogenate was then oscillated at 4 °C for 30 min and centrifuged at 1200× *g* and 4 °C for 5 min. The resulting supernatant, representing the cytoplasmic fraction, was carefully aspirated and transferred to a pre-cooled sterile centrifuge tube for subsequent analysis. The remaining pellet was reconstituted in ice-cold PBS and centrifuged at 2000× *g* for 5 min at 4 °C, followed by complete removal of the supernatant. The remaining pellet, representing the nuclear fraction, was resuspended in 200 μL of preservation buffer B, and the protein concentration was determined using a BCA assay kit. The nuclear translocation of Nrf2 in the heart was measured as described in Section 2.9.1.

### 2.10. Histopathological Observation

#### 2.10.1. HE (Hematoxylin and Eosin) Staining

The duodenum, jejunum, and ileum tissues were immersed in 10% neutral formalin and rinsed under running water for 24 h. Subsequent dehydration, embedding, and sectioning were performed using an automatic tissue processor, yielding sections with a thickness of 4 μm. The sections were then baked at 60 °C for 4 h. Dewaxing of the sections was carried out using xylene I, xylene II, benzene-alcohol, and a graded series of ethanol (100% ethanol I, 100% ethanol II, 90% ethanol I, 90% ethanol II, and 80% ethanol). The tissue sections were stained with hematoxylin for 5 min and subsequently washed with continuous running deionized water for 15 min to ensure complete removal of excess stain. Differentiation was performed using a differentiating solution, and the sections were rinsed again with running water for 15 min. Eosin staining was performed for 15 min, after which the sections were gradually dehydrated in ascending concentrations of alcohol and cleared. Finally, the sections were mounted. A digital slice scanner (DX1; Shandong, China) was used to collect pictures and observe the damage to the duodenum, jejunum, and ileum tissues.

#### 2.10.2. AB-PAS Staining (Alcian Blue-Periodic Acid-Schiff Staining)

The tissue sections underwent standard dewaxing procedures, followed by a 15-min rinse under continuous running deionized water. Subsequently, the sections were stained with Alcian Blue for 15 min, gently washed with distilled water, treated with 1% periodic acid solution for 5 min, and an additional 10-min distilled water rinse. The sections were then incubated with Schiff’s reagent for 15 min and thoroughly rinsed under running deionized water for 10 min. Finally, the sections were processed through a graded ethanol series for dehydration, cleared in xylene, and permanently mounted using a synthetic resin. Images were collected using a digital slice scanner (DX1; Shandong, China), and the number of goblet cells and mucin secretion in the duodenum, jejunum, and ileum were observed.

### 2.11. High-Throughput Sequencing Analysis of the Gut Microbiota

Upon completion of the experimental protocol, fecal specimens were collected from the rat subjects. Genomic DNA was extracted using a kit with bead-beating lysis (3 × 60 s) and RNase A treatment, followed by quality verification. The V3–V4 region of bacterial 16S rRNA genes was amplified using primers 341F/805R under standardized PCR conditions, followed by AMPure XP bead purification and Illumina Nextera XT indexing. The libraries were sequenced using an Illumina NovaSeq 6000 platform. Raw FASTQ data were processed in QIIME2 through (1) quality filtering and denoising (DADA2 for ASV generation), (2) taxonomic classification against the SILVA 138 database, and (3) diversity analyses (alpha: Shannon/Chao1; beta: weighted UniFrac PCoA). Differential microbial analysis was performed using one-way ANOVA.

### 2.12. Statistical Analysis

The experimental results are expressed as the mean ± standard error (SEM). Statistical analyses were performed using SPSS software (version 26.0; IBM Corp., Armonk, NY, USA). One-way analysis of variance (ANOVA) was used for multiple comparisons, while independent two-sample *t*-tests were applied for comparisons between two groups. Graphs were generated using GraphPad Prism 9 software. A *p*-value of <0.05 was considered statistically significant.

## 3. Results

### 3.1. Effects of Different Rewarming Durations on Heat-Stressed Rats

**Figure 1:**
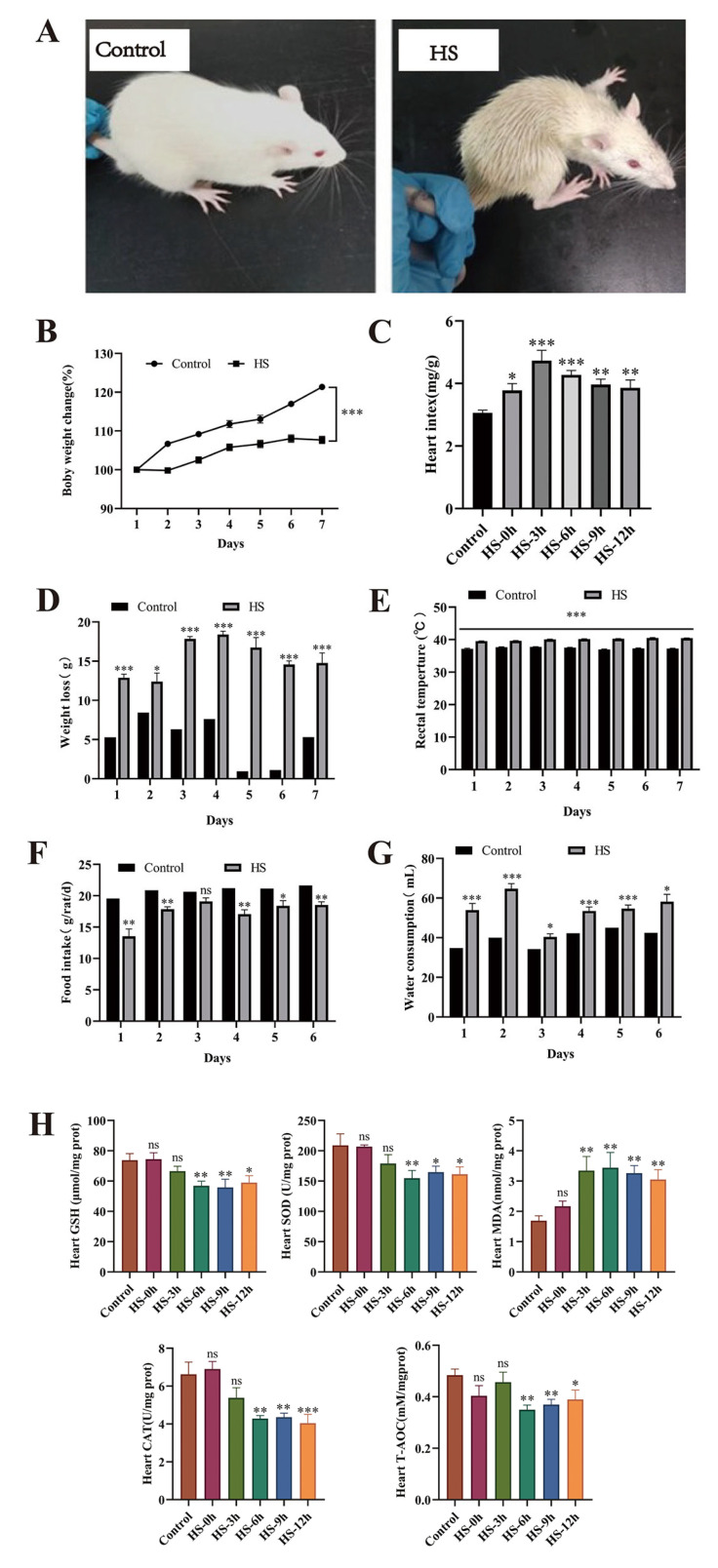
Effects of different recovery times on heat-stressed rats. (**A**) Behavioral changes in heat-stressed rats; (**B**) Body weight change rate; (**C**) Changes in cardiac index at different recovery time points; (**D**) Body weight loss before and after heat stress; (**E**) Changes in rectal temperature; (**F**) Changes in food intake; (**G**) Changes in water intake; (**H**) Effects of heat stress on the levels of GSH, SOD, MDA, CAT, and T-AOC in rat hearts. * *p* < 0.05, ** *p* < 0.01, *** *p* < 0.001 compared to the control group (n ≥ 5/group); “ns” indicates no statistically significant difference (*p* > 0.05).

We observed and analyzed the behavioral and physiological changes and oxidative stress indicators in rats during heat stress. The results revealed that heat-stressed rats exhibited rough and disheveled fur, increased respiratory rates, protruding eyeballs, and reddish limbs. In the early stage, they showed lethargy and stillness, followed by restless behavior in the middle stage, and finally, they lay sprawled with their limbs extended (Figure 1A). On the 7th day after heat stress, compared with the control group, the weight change rate of rats in the HS group was significantly decreased (*p* < 0.05) (Figure 1B), while the heart index of rats at different rewarming time points was significantly increased (*p* < 0.05) (Figure 1C). The average weight loss of the rats before and after heat stress was 18.46 g, and the average weight loss of the control group was 4.08 g (Figure 1D). Heat stress significantly increased the rectal temperature of rats, with body temperature increasing by more than 40 °C (*p* < 0.05) (Figure 1E). Compared with the control group, the water intake of heat-stressed rats increased significantly, while the feed intake decreased significantly (*p* < 0.05) (Figure 1F,G). Analysis of oxidative stress indicators revealed that, compared to the control group, the amounts of SOD, GSH, T-AOC, and CAT in the hearts of heat-stressed rats gradually decreased with prolonged recovery time, becoming particularly significant at 6–12 h (*p* < 0.05). Conversely, MDA levels increased with recovery time, peaking at 6 h (Figure 1H).

### 3.2. Protective Effects of Sheng Mai San on Heat-Stressed Rats

**Figure 2:**
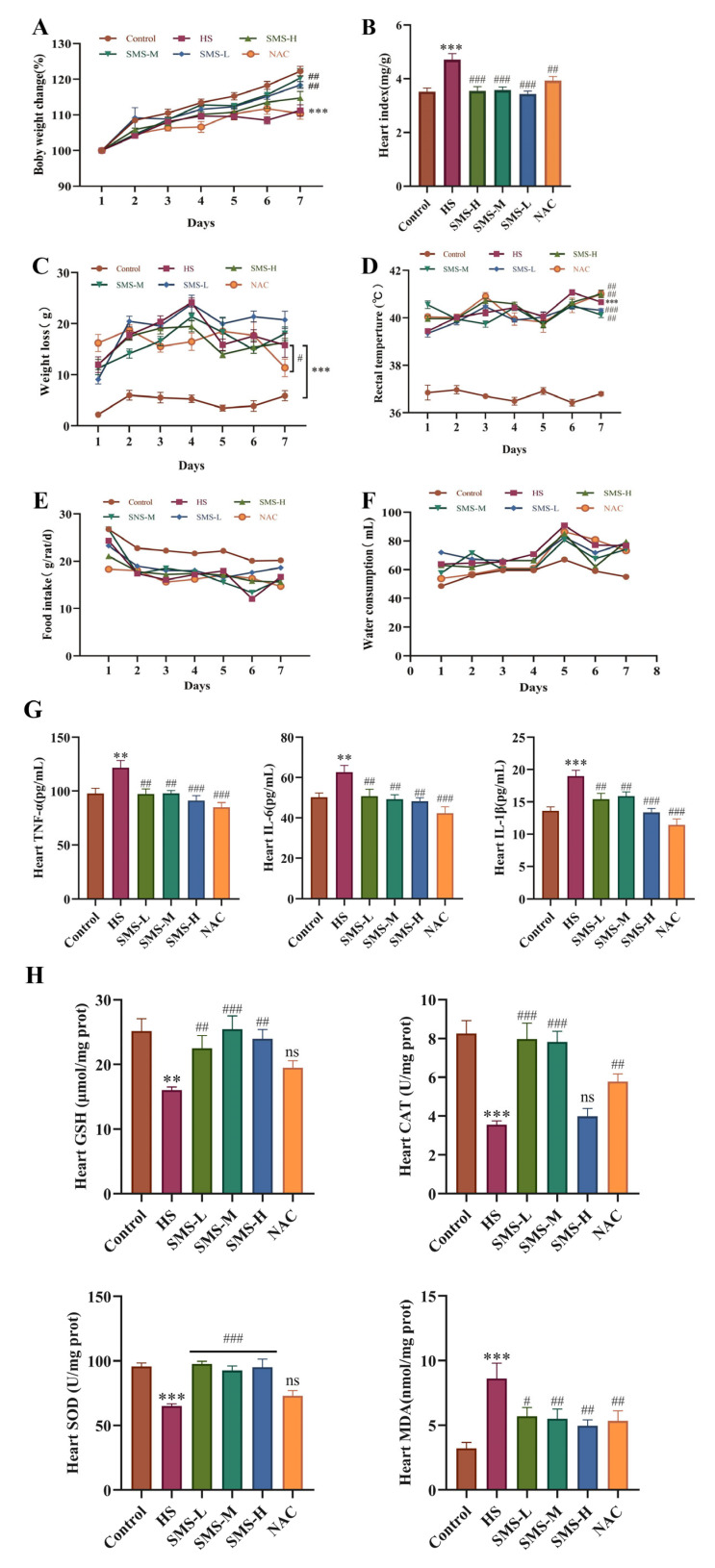
Intervention effects of Sheng Mai San on heat-stressed rats. (**A**) Body weight change rate; (**B**) Cardiac index; (**C**) Body weight loss; (**D**) Rectal temperature; (**E**) Food intake; (**F**) Water intake; (**G**) Effects of Sheng Mai San on the levels of TNF-α, IL-6, and IL-1β in the hearts of heat-stressed rats; (**H**) Effects of Sheng Mai San on the levels of GSH, CAT, SOD, and MDA in the hearts of heat-stressed rats. Compared with the control group, ** *p* < 0.01, *** *p* < 0.001; compared with the HS group, # *p* < 0.05, ## *p* < 0.01, ### *p* < 0.001, (n ≥ 5/group); “ns” indicates no statistically significant difference (*p* > 0.05).

We intervened in heat-stressed rats using Sheng Mai San. The results showed that after heat stress, the body weight change rate significantly decreased, cardiac index significantly increased, body weight loss significantly increased, and rectal temperature significantly increased. Following 7 days of intervention with Sheng Mai San and NAC, all indicators showed varying degrees of improvement, with the low and medium doses of Sheng Mai San demonstrating better efficacy (Figure 2A–D). However, no appreciable variations in food intake and water consumption were observed among the different groups after heat stress (*p* > 0.05) (Figure 2E,F). Analysis of inflammatory factors and oxidative stress indicators revealed that, compared to the control group, the levels of IL-6, IL-1β, and TNF-α in the hearts of heat-stressed rats were significantly elevated (*p* < 0.01), while GSH, CAT, and SOD levels were significantly reduced (*p* < 0.01). Additionally, MDA levels were significantly increased (*p* < 0.01). Following intervention with Sheng Mai San and NAC, all indicators showed varying degrees of improvement, with the low- and medium-dose groups of Sheng Mai San demonstrating better efficacy (Figure 2G,H).

### 3.3. Effects of Sheng Mai San on Keap1-Nrf2 Pathway Protein Levels in the Hearts of Heat-Stressed Rats

**Figure 3:**
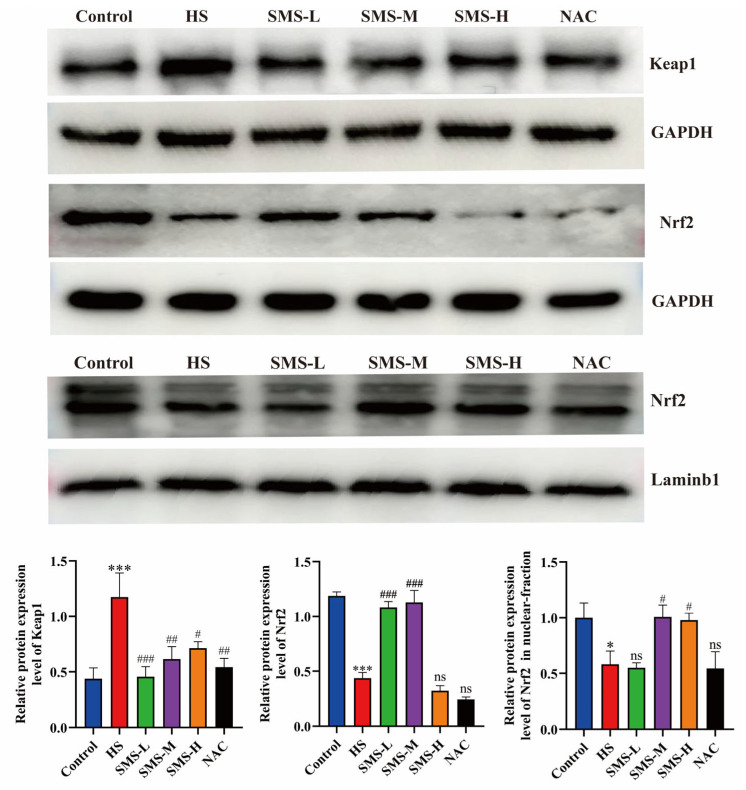
Effects of Sheng Mai San on the Keap1-Nrf2 pathway in the hearts of heat-stressed rats. Compared with the control group, * *p* < 0.05, *** *p* < 0.001; compared with the HS group, # *p* < 0.05, ## *p* < 0.01, ### *p* < 0.001, (n ≥ 3/group); “ns” indicates no statistically significant difference (*p* > 0.05).

To further demonstrate the impact of heat stress on cardiac oxidative stress in rats, we used Western blotting to detect proteins related to the Keap1-Nrf2 pathway. The results showed that Keap1 protein expression was significantly increased compared to the control group (*p* < 0.01), while Nrf2 protein expression and the nuclear content of Nrf2 were significantly decreased (*p* < 0.05) after heat stress treatment. Following intervention with Sheng Mai San and NAC, these indicators showed varying degrees of improvement, with the medium-dose Sheng Mai San group exhibiting the most pronounced effects (Figure 3).

### 3.4. Effects of Sheng Mai San on Pathological Changes in the Small Intestine of Heat-Stressed Rats

**Figure 4:**
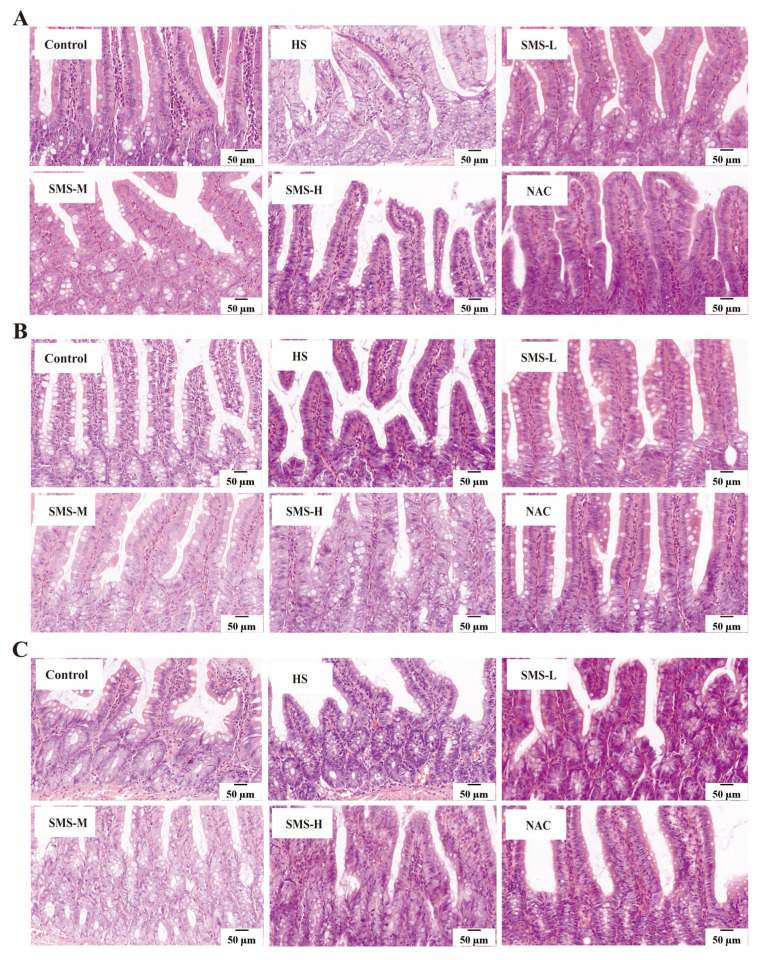
Effects of Sheng Mai San on pathological changes in the small intestine of heat-stressed rats (×400). (**A**) HE staining of duodenal pathology; (**B**) HE staining of jejunal pathology; (**C**) HE staining of ileal pathology (n ≥ 3/group).

**Figure 5:**
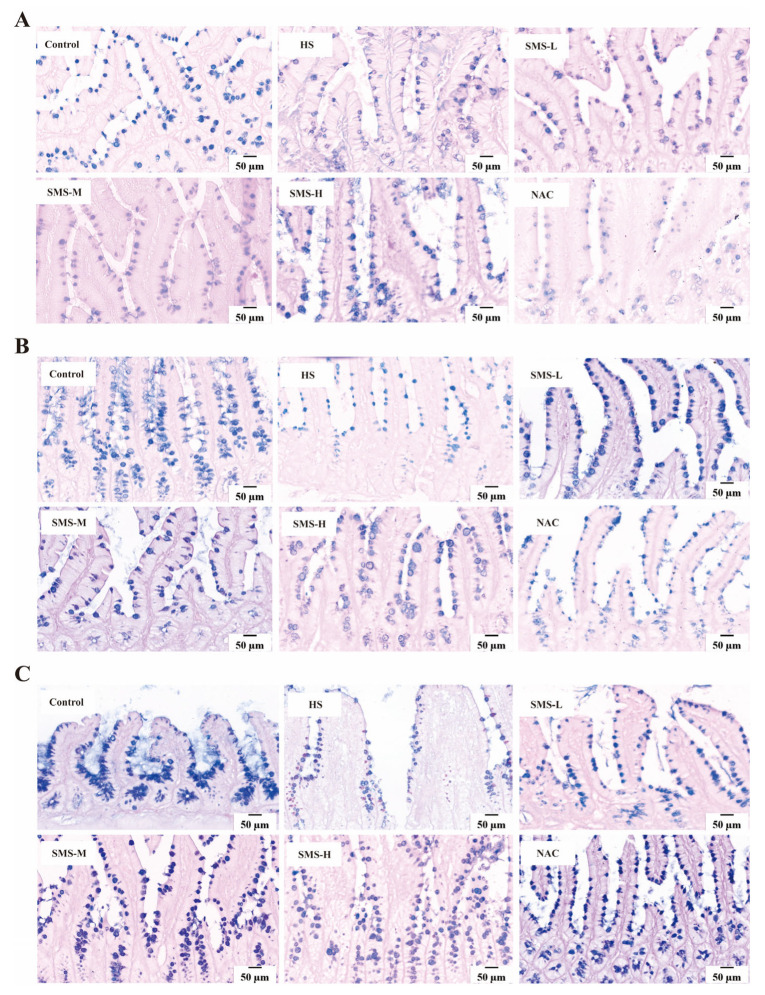
Effect of Sheng Mai San on Goblet Cell Populations in the Small Intestine of Heat-Stressed Rats. (**A**) Alterations in duodenal goblet cell density; (**B**) Variations in jejunal goblet cell counts; (**C**) Modifications in ileal goblet cell abundance (n ≥ 3/group).

To determine how Sheng Mai San affects the small intestine of heat-stressed rats, we observed the pathological changes and the number of goblet cells in the small intestine of rats using HE and AB-PAS staining. The results showed that there were no significant adhesions or damage in the small intestine of the control group rats, and the intestinal villi were intact and arranged regularly and tightly. In heat-stressed rats, however, some villi in the duodenum were shed, edema was severe, and the structure of some intestinal epithelial cells was unclear. Severe villus shedding was observed in the jejunum, accompanied by partial edema. In the ileum, some villi were shed, and mild congestion and edema were present. After intervention with Sheng Mai San, various pathological symptoms improved, with better effects observed in the low- and medium-dosage groups of Sheng Mai San (Figure 4). AB-PAS staining results showed that rats under heat stress had significantly fewer goblet cells in the ileum, jejunum, and duodenum in comparison to the control group. However, as a result of Sheng Mai San and NAC’s intervention, the number of goblet cells increased in all intestinal segments, with better effects observed in the low- and medium-dosage groups of Sheng Mai San (Figure 5).

### 3.5. Effects of Sheng Mai San on Gut Microbiota Diversity in Heat-Stressed Rats

**Figure 6:**
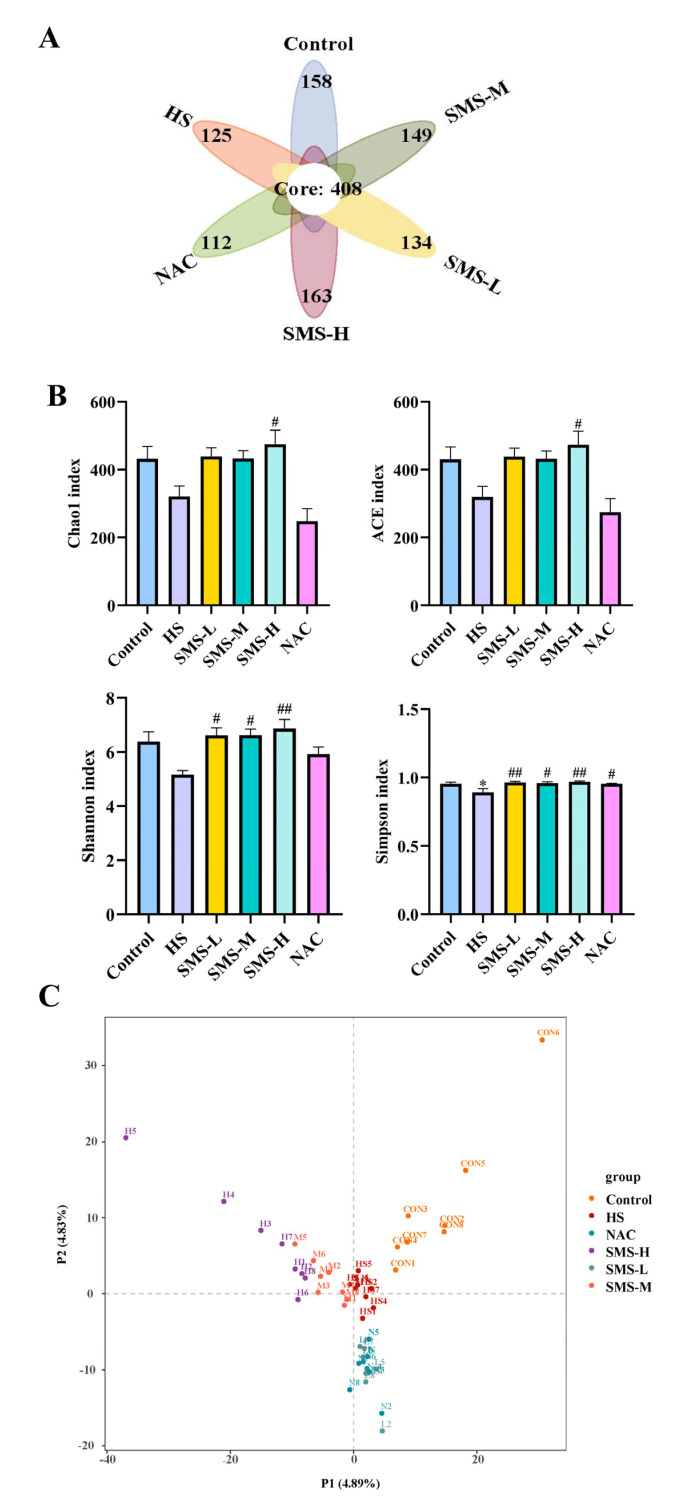
Effects of Sheng Mai San on the diversity of gut microbiota in heat-stressed rats. (**A**) Venn diagram showing the number of gut microbiota genes in different treatment groups; (**B**) Effects of Sheng Mai San on the alpha-diversity of gut microbiota in heat-stressed rats; (**C**) PLS-DA analysis of different treatment groups. Compared with the control group, * *p* < 0.05; compared with the HS group, # *p* < 0.05, ## *p* < 0.01, (n ≥ 5/group).

We employed high-throughput sequencing to analyze the gut microbiota of the rats. The results revealed that the control group, heat stress group, Sheng Mai San intervention group, and NAC intervention group shared a total of 408 common operational taxonomic units (OTUs). Compared to the control group, the heat-stressed group exhibited 125 unique OTUs (Operational Taxonomic Units), whereas the low-, medium-, and high-dose Sheng Mai San intervention groups had 134, 149, and 163 unique OTUs, respectively, indicating the influence of Sheng Mai San on heat-stressed rats. The NAC intervention group had 112 unique OTUs (Figure 6A). Analysis of alpha-diversity indices revealed that, compared to the control group, the Chao1, ACE, and Shannon indices exhibited a decreasing trend in heat-stressed rats, although the differences were not statistically significant (*p* > 0.05). However, the Simpson index was significantly reduced (*p* < 0.05). Following intervention with Sheng Mai San, all indices demonstrated an increasing trend, with the high-dose Sheng Mai San group showing the most pronounced improvement. In contrast, NAC only upregulated the Simpson index (Figure 6B). The control group and the HS group could be clearly distinguished from one another through PLS-DA (Partial Least Squares Discriminant Analysis), and also demonstrated distinct separations among the low-dose, high-dose Sheng Mai San groups, the NAC group, and the HS group (Figure 6C).

### 3.6. Effects of Sheng Mai San on Differential Gut Microbiota in Heat-Stressed Rats

**Figure 7:**
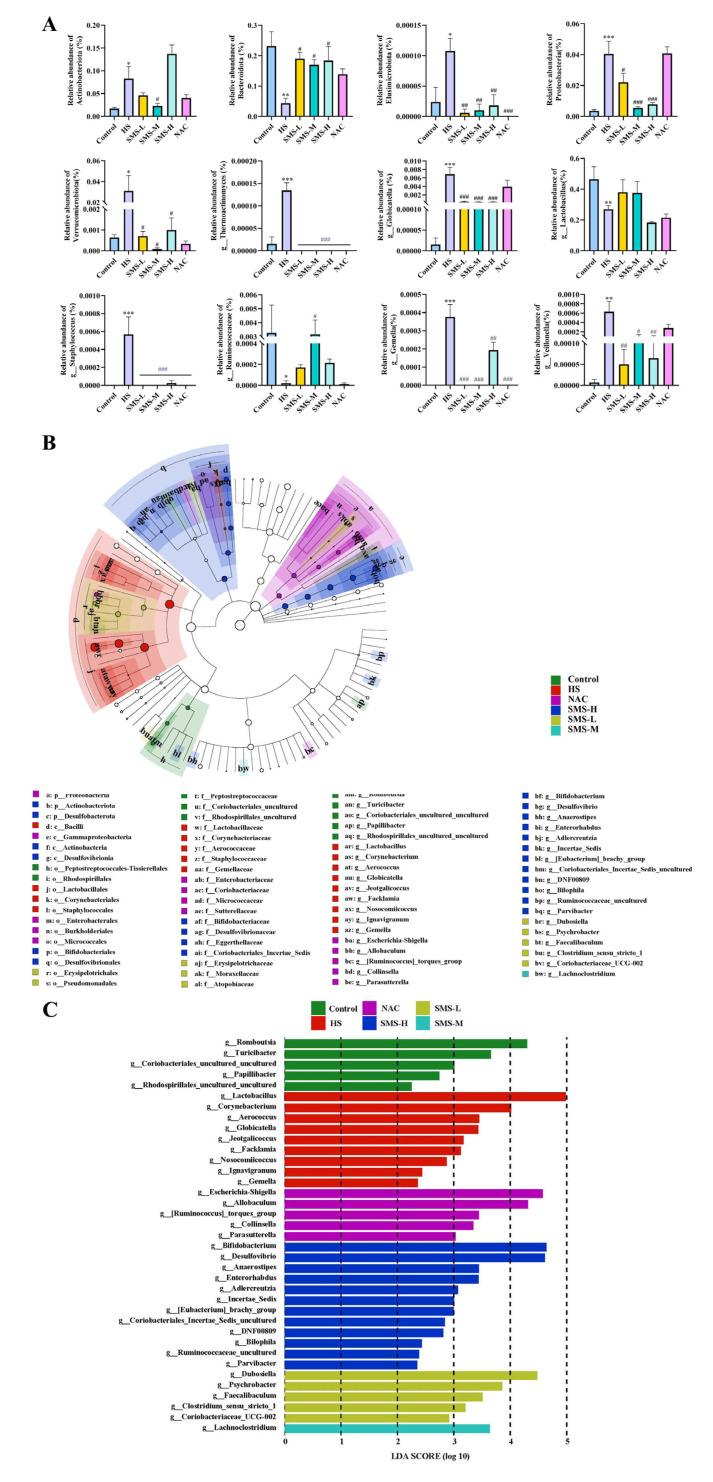
Effects of Sheng Mai San on differential gut microbiota in heat-stressed rats. (**A**) Analysis of differential microbiota at the phylum and genus levels in different treatment groups; (**B**) LEfSe (Linear Discriminant Analysis (LDA) Effect Size) analysis of different treatment groups, where an LDA score greater than 3 was considered statistically significant; (**C**) Cladogram of different treatment groups. Compared with the control group, * *p* < 0.05, ** *p* < 0.01, *** *p* < 0.001; compared with the HS group, # *p* < 0.05, ## *p* < 0.01, ### *p* < 0.001, (n ≥ 5/group).

Analysis of differential microbiota at the phylum and genus levels revealed that, compared to the control group, the abundances of Actinobacteriota, Elusimicrobiota, Proteobacteria, and Verrucomicrobiota were significantly increased (*p* < 0.05), whereas the abundance of Bacteroidota in the HS group was substantially reduced (*p* < 0.01). After intervention with Sheng Mai San, these phyla showed varying degrees of recovery (*p* < 0.05), with the medium-dose Sheng Mai San group exhibiting the most significant improvement, whereas the NAC intervention showed limited effects (Figure 7A). At the genus level, the abundances of *g_Globicatella*, *g_Thermoactinomyces*, *g_Staphylococcus*, *g_Gemella*, and *g_Veillonella* were substantially increased (*p* < 0.05), while *g_Lactobacillus* and *g_Ruminococcaceae* were significantly decreased in the heat stress group. Following Sheng Mai San intervention, these genera showed recovery, with the medium-dose Sheng Mai San group demonstrating the best efficacy (Figure 7A).

Linear Discriminant Analysis (LDA) Effect Size (LEfSe) was used to analyze and compare the microbial composition among the different treatment groups and identify specific bacterial taxa for each group (Figure 7B,C). The results revealed that the control group was enriched with three key genera: *g_Romboutsia*, *g_Turicibacter*, and *g_Coriobacteriales_uncultured*, which primarily belonged to the phyla Firmicutes and Actinobacteriota. In contrast, the heat stress group was enriched with six key genera: *g__Lactobacillus*, *g_Corynebacterium*, *g_Aerococcus*, *g_Globicatella*, *g_Jeotgalicoccus*, and *g_Facklamia*, which primarily belonged to the phyla Firmicutes and Actinobacteriota. The low-, medium-, and high-dose Sheng Mai San groups restored 4, 1, and 6 key genera with discriminatory features, respectively, primarily belonging to the phyla Firmicutes, Actinobacteria, and Proteobacteria. The NAC group primarily enriched five key genera within the phyla Firmicutes, Actinobacteria, and Proteobacteria.

## 4. Discussion

Heat stress can disrupt thermoregulatory mechanisms, leading to a series of physiological and behavioral changes [24]. Our study found that heat stress resulted in reduced body weight, food intake, and water consumption in rats, which may be associated with imbalances in energy metabolism and increased protein breakdown [25]. Under high-temperature conditions, rats reduce heat-producing activities to lower their body temperature, and since feeding and digestion processes are accompanied by increased heat production, rats instinctively decrease their food intake. Reduced food intake leads to insufficient energy intake, while the metabolic rate increases to dissipate heat, resulting in higher energy expenditure. Additionally, high temperatures can diminish thirst sensations in rats, suppressing their drinking behavior. Furthermore, heat stress, a common environmental stressor, has been shown to significantly affect multiple organ systems, with the heart being particularly vulnerable due to its central role in maintaining circulatory and metabolic homeostasis. Studies have indicated that heat stress-induced cardiac injury primarily occurs within 6–12 h of heat stress exposure, a critical time window characterized by significant changes in cardiac function and biochemical markers. Specifically, this period is marked by a notable increase in the cardiac index, decline in key antioxidants (such as GSH, SOD, CAT, and T-AOC), and elevated levels of the lipid peroxidation product MDA. These changes collectively suggest that oxidative stress and impaired antioxidant defense mechanisms play pivotal roles in heat-stress-induced cardiac injury. It was found that during the early stages (within 6 h) of heat stress, the cardiac index increased significantly. This elevation may reflect a compensatory mechanism to meet the heightened metabolic demands and increased circulatory load caused by heat stress (Gathiram et al., 1987) [26]. However, this compensatory mechanism is limited, and prolonged elevation of the cardiac index can lead to myocardial injury and deterioration of cardiac function [26]. Antioxidant enzymes, which are capable of scavenging or neutralizing free radicals and reactive oxygen species, play a major role in maintaining intracellular redox balance and protecting against oxidative damage. Among these, SOD, CAT, and GPx are critical components of the antioxidant enzyme system [27]. The experimental results revealed that heat stress exposure induced a significant suppression of SOD and GPx enzymatic activities in porcine myocardial tissue, concurrent with a marked elevation in MDA levels, indicative of enhanced lipid peroxidation (McCord et al., 1969) [28]. Although heat stress rapidly induces excessive generation of ROS, the peak effects of oxidative stress are often delayed. The investigation revealed that 6 h post-heat stress exposure, significant elevations in LDH levels were observed in both serum and myocardial tissues of murine models, concomitant with a pronounced reduction in SOD activity in myocardial tissues. Parallel in vitro experiments substantiated these findings, demonstrating that heat-stressed cells exhibited marked alterations in cellular viability, antioxidant capacity (as reflected by SOD and GSH activities), ROS accumulation, and LDH release (Wang et al., 2024) [29], consistent with our research findings. These changes collectively demonstrate that oxidative stress and impaired antioxidant defense mechanisms play critical roles in heat stress-induced cardiac injury.

According to traditional Chinese (veterinary) medicine, the heart governs blood circulation and supports small intestine function through heart yang (warming) and heart blood (nourishing). The small intestine digests food, absorbs nutrients, and transports them to the heart for blood production. These organs are interconnected as exterior-interior pairs, meaning that dysfunction in one can affect the other. For example, heart fire can cause small intestine heat symptoms, while small intestine heat may lead to heart-related issues [30]. Sheng Mai San, a classic formulation for replenishing qi and nourishing yin, is clinically used to treat cardiovascular diseases, diabetes, liver injury, heatstroke, and shock [31,32,33,34,35,36]. However, research on Sheng Mai San for mitigating heat stress injury is limited. In our study, we intervened in heat-stressed rats with Sheng Mai San and found that it reduced the cardiac index, thereby alleviating the cardiac load. It significantly increased the levels of GSH, SOD, and CAT, while decreasing MDA content in the heart. Additionally, Sheng Mai San reduced the levels of IL-1β, IL-6, and TNF-α in the heat-stressed hearts. These results indicate that Sheng Mai San effectively mitigates heat-stress-induced cardiac injury by enhancing antioxidant defense mechanisms and suppressing inflammatory responses. The Keap1-Nrf2 pathway is a critical regulatory mechanism that enables cells to combat oxidative stress. Under normal conditions, Keap1 binds to Nrf2, promoting its ubiquitination and degradation, thereby maintaining low levels of Nrf2. The heart is a high-energy-demand organ that is highly sensitive to oxidative stress. Heat stress leads to the accumulation of ROS in cardiomyocytes, triggering apoptosis, inflammation, and fibrosis. According to our research, heat stress inhibits the expression of the Keap1-Nrf2 pathway in the heart. Additionally, we found that Sheng Mai San intervention activated the Keap1-Nrf2 pathway, promoting the nuclear translocation of Nrf2, thereby enhancing antioxidant capacity and mitigating cardiac injuries.

Heat stress not only directly causes cardiac injury but may also further induce small intestinal damage through the “heart-gut axis” mechanism. Cardiac injury induced by heat stress leads to decreased cardiac function, which subsequently affects systemic blood circulation, resulting in insufficient intestinal blood perfusion and triggering intestinal ischemia and hypoxia [37]. Furthermore, inflammatory cytokines and oxidative stress mediators released during cardiac injury can systemically disseminate through the circulatory system, thereby exacerbating intestinal mucosal damage and potentiating pathological alterations in the small intestine [38]. According to our research, the duodenum, jejunum, and ileum of rats can sustain pathological damage from heat stress. This damage is mostly characterized by edema, villus shedding, unclear epithelial cell shape, and a decrease in goblet cell count. However, intervention with Sheng Mai San significantly mitigated heat-stress-induced pathological damage in the rat small intestine. Using HE staining, Zhang et al. observed that heat stress caused severe damage to the small intestinal epithelium. Compared to the control tissues, heat-stressed tissues exhibited detachment of epithelial cells from the basement membrane at the villus tips. At higher magnification, vacuolization of epithelial cells was also observed, with severe cases showing exposed lamina propria [39]. This disruption of barrier function provides an opportunity for the colonization and proliferation of pathogenic bacteria while potentially inhibiting the growth of beneficial bacteria, leading to intestinal dysbiosis [40]. Our results demonstrated that heat-stressed rats exhibited significant reductions in microbial alpha-diversity indices, including Chao1, ACE, Shannon, and Simpson indices, suggesting that heat stress may reduce the diversity and richness of the gut microbiota in rats. Through differential microbiota analysis, it was revealed that heat stress resulted in increased expression of harmful bacteria, such as *g_Globicatella*, *g_Thermoactinomyces*, *g_Staphylococcus*, *g_Gemella*, and *g_Veillonella*, and decreased expression of *g_Lactobacillus* and *g_Ruminococcaceae*. However, these bacterial populations were partially restored after intervention with Sheng Mai San. The overproliferation of *g_Globicatella*, *g_Gemella*, *g_Staphylococcus*, and *g_Veillonella* may exacerbate systemic inflammation by producing pro-inflammatory metabolites, thereby promoting the development of cardiac diseases, such as atherosclerosis and endocarditis. These findings reveal that cardiac and intestinal diseases mutually influence each other, and that there is a close association between gut microbiota and cardiac health. This underscores the importance of maintaining gut microecological balance for the prevention and treatment of cardiovascular diseases and provides new research directions for future interventions in cardiovascular diseases through the modulation of gut microbiota. Furthermore, in patients with inflammatory bowel disease (IBD), *g_Staphylococcus* and *g_Veillonella* are significantly elevated, and they can disrupt intestinal epithelial cells and the mucus layer, increasing intestinal permeability and leading to bacterial and endotoxin translocation, which further exacerbates systemic inflammation [41,42]. *g_Lactobacillus*, a crucial probiotic in the gut, plays a vital role in maintaining intestinal barrier function, exerting antioxidant effects, regulating immune responses, and improving microbial balance [43]. We observed a significant reduction in *g_Lactobacillus* abundance in heat-stressed rats. Heat stress also led to a notable decrease in *g_Ruminococcaceae*, a key fiber-degrading bacterium in the gut that ferments dietary fiber to produce SCFAs, such as butyrate, acetate, and propionate. These microbial metabolites are vital for maintaining intestinal homeostasis and the integrity of the mucosal barrier. Future research should further explore the specific mechanisms of *g_Thermoactinomyces* under heat stress conditions and its impact on the host. These findings demonstrate that Sheng Mai San alleviates heat stress-induced cardiac injury, small intestinal pathological changes, and gut microbiota dysbiosis through multi-target and multi-pathway mechanisms, providing a scientific basis for the application of traditional Chinese medicine in modern medicine.

## 5. Conclusions

Heat stress-induced cardiac injury primarily occurs within 6–12 h after the cessation of heat stress, as indicated by an increase in the cardiac index and a decrease in the levels of cardiac antioxidant enzymes. Sheng Mai San ameliorates heat stress-induced cardiac hypertrophy, enhances the expression of antioxidant enzymes (GSH, SOD, and CAT), activates the Keap1-Nrf2 pathway in the heart, and restores oxidative stress balance. Additionally, Sheng Mai San alleviates small intestinal injury, increases the richness and diversity of gut microbiota, and improves symptoms of gut microbiota dysbiosis.

## Data Availability

The datasets used and/or analyzed during the current study are available from the corresponding author upon reasonable request.

## Abbreviations

Sheng Mai San, SMS; heat stress, HS; reactive oxygen species, ROS; catalase, CAT; superoxide dismutase, SOD; glutathione, GSH; total antioxidant capacity, T-AOC; malondialdehyde, MDA; operational taxonomic units, OTUs; Nuclear factor erythroid 2-related factor 2, Nrf2; Kelch-like ECH-associated protein 1, Keap1; N-acetylcysteine, NAC; Hematoxylin and Eosin, HE; Alcian Blue-Periodic Acid-Schiff, AB-PAS.
